# Bond-length dependence of attosecond ionization delays in O_2_ arising from electron correlation to a shape resonance

**DOI:** 10.1126/sciadv.adl3810

**Published:** 2024-03-27

**Authors:** Daniel Hammerland, Thomas Berglitsch, Pengju Zhang, Tran Trung Luu, Kiyoshi Ueda, Robert R. Lucchese, Hans Jakob Wörner

**Affiliations:** ^1^ ETH Zürich, Laboratorium für Physikalische Chemie, Zürich, Switzerland.; ^2^ Department of Physics, The University of Hong Kong, SAR Hong Kong, China.; ^3^ Department of Chemistry, Tohoku University, Sendai, Japan.; ^4^ Lawrence Berkeley National Laboratory, Berkeley, USA.

## Abstract

We experimentally and theoretically demonstrate that electron correlation can cause the bond-length sensitivity of a shape resonance to induce an unexpected vibrational state–dependent ionization delay in a nonresonant channel. This discovery was enabled by a high-resolution attosecond-interferometry experiment based on a 400-nm driving and dressing wavelength. The short-wavelength driver results in a 6.2–electron volt separation between harmonics, markedly reducing the spectral overlap in the measured interferogram. We demonstrate the promise of this method on O_2_, a system characterized by broad vibrational progressions and a dense photoelectron spectrum. We measure a 40-attosecond variation of the photoionization delays over the X^2^Π*_g_* vibrational progression. Multichannel calculations show that this variation originates from a strong bond-length dependence of the energetic position of a shape resonance in the $b^{4}\Sigma_{g}^{-}$ channel, which translates to the observed effects through electron correlation. The unprecedented energy resolution and delay accuracies demonstrate the promise of visible-light–driven molecular attosecond interferometry.

## Teaser

Electron correlation to a shape resonance causes a 40-attosecond change of photoionization delays over a vibrational progression.

## INTRODUCTION

Early photoelectron measurements with tunable light sources found local maxima in the photoionization cross sections of molecules just above their ionization thresholds. These maxima were later identified as “shape resonances” (*1*–*4*), which have received substantial attention in frequency-domain measurements in the extreme-ultraviolet (XUV) and soft x-ray domains. These investigations revealed that small variations in bond length could markedly alter the resonance central energy and width (*5*–*7*). Furthermore, it was discovered that these single-channel phenomena could be imprinted onto otherwise nonresonant ionization channels through electron correlation (*8*–*12*). With the advent of attosecond spectroscopy, shape resonances attracted renewed interest because their ultrashort lifetimes could be accessed directly for the first time. Attosecond time–resolved measurements, highlighted in the 2023 Nobel Prize in Physics (*13*), found that shape resonances could induce photoionization delays of up to hundreds of attoseconds compared to nonresonant ionization (*14*). Subsequent studies have shown that these large delays vary strongly with the emission angle and the bond length (*15*–*20*). Further studies showed that the large delays from shape resonances can be imprinted onto other ionization channels through interchannel coupling (*21*). However, the electron-correlation–mediated effect of shape resonances on the vibrational-state dependence of photoionization delays in other channels has been neither observed nor theoretically discussed.

In this work, we observe and rationalize the bond-length sensitivity of a molecular shape resonance imprinted on other nonresonant ionization channels through electron correlation, particularly photoionization channel coupling. We measure a variation of the photoionization delays of ∼40 as across the resolved vibrational states (*v* = 0 to 4) of the X^2^Π*_g_* state of $O_{2}^{+}$ , an unexpectedly large range given the absence of shape resonances in this channel at these energies. Through comparison with single- and multi-channel ab initio photoionization calculations, we show that the experimental observations are a direct signature of a shape resonance in a different channel, the $b^{4}\Sigma_{g}^{-}$ channel, which is imprinted onto the X^2^Π*_g_* channel through the effects of electron correlation. This work thus uncovers a mechanism that can cause large variations of photoionization delays across a vibrational progression.

The discovery reported in this work was enabled by an innovative attosecond-interferometry scheme based on 400-nm high harmonic generation (HHG) and dressing. There are experimental compromises inherent to this shorter-wavelength–based experiment; however, they are favorable for a broad range of applications. The scaling laws associated with the strong-field approximation of HHG predict that the high-harmonic efficiency scales roughly according to λ^−6^ (*22*), meaning that a reduction in the generating wavelength by a factor of 2 compared to an 800-nm–based attosecond-interferometry experiment results in a substantial increase in the XUV flux. This improves the statistics of experiments and allows for the measurement of relatively weak phenomena. It does bring with it the caveat that the reachable photon energies are decreased, as the high-harmonic cutoff energy scales according to λ^2^ (*22*). However, a majority of attosecond interferometry experiments are performed at relatively low XUV photon energies, making this compromise beneficial for many applications.

The period of the oscillations that are measured during a 400-nm–based experiment is reduced from 1.333 to 0.667 fs. While this decreased delay window might limit the dynamics that can be resolved with this technique to the sub-femtosecond regime, it also means that the numerical error resulting from the analysis is reduced, as the period of the phases extracted is reduced by a factor of 2 (*23*), allowing for higher precision on smaller measured delays.

The second-harmonic-generation process also introduces a relatively simple method of altering the optical bandwidths in the experiment. By varying the crystal thickness, one can coarsely adjust the spectral broadness to either increase the flux or decrease the optical bandwidth to improve the spectral resolution.

Perhaps the greatest benefit of the 400-nm–based attosecond interferometry is the 6.2-eV separation between the generated harmonics. Spectral overlap has been a long-standing issue for attosecond interferometry. While there are fitting procedures that can take into account the effects of overlapped spectral features (*24*), decongesting the spectra simplifies the analysis process and allows for higher fidelity delay measurements on more complex samples.

## RESULTS

**Fig. 1.**
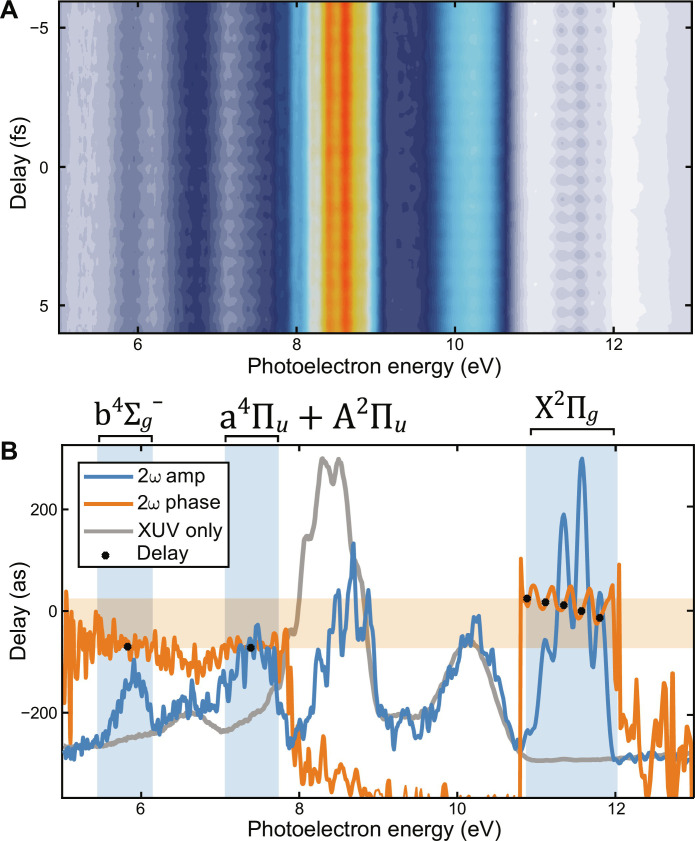
Experimental data and delay extraction. Plot of the attosecond interferogram (**A**) measured in O_2_. (**B**) The 2ω amplitude (blue), 2ω phase (orange), XUV-only spectrum (gray), and the extracted delays. The blue-shaded areas highlight the positions of sideband 8 for the X^2^Π*_g_*, a^4^Π*_u_*/A^2^Π*_u_*, and $b^{4}\Sigma_{g}^{-}$ states. The orange shading emphasizes the range of the delays measured, which amounts to roughly 100 as.

The attosecond interferogram, shown in Fig. 1A, was acquired by taking 128 time steps over ~18 full periods of the 2ω oscillations of the dressing field. Each scan required roughly 40 min for acquisition. The data analysis is performed by taking a Fourier transform along the time-delay axis and isolating the 2ω amplitude and phase. The phase is divided by the period of the oscillations to get the delay. The amplitude and delay are shown in Fig. 1B. The spectral separation and resolution afforded by the 400-nm HHG and dressing scheme allow for simple identification of the sidebands and main peaks by comparing the XUV-only spectrum (gray), to the 2ω amplitude (blue). The blue-shaded areas in Fig. 1B emphasize the clearly separated sidebands and main peaks. The extracted delays are found by taking the 2ω amplitude weighted average delay over each of the vibrationally resolved peaks. The extracted phases are shown as black spots in Fig. 1B. The orange shading accentuates the range of the observed delays. The *v* = 1, the largest-amplitude vibrational peak in the X^2^Π*_g_* state, serves as the reference for all the delays. The delays from *v* = 0 to *v* = 4 are −13.4 ± 1.0 as, 0/ref, 11.5 ± 1.8 as, 17.2 ± 1.9 as, and 24 ± 7 as. The delay between the X^2^Π*_g_*, *v* = 1 and the a^4^Π*_u_* + A ^2^Π*_u_* and $b^{4}\Sigma_{g}^{-}$ states amount to −72 ± 3 as and −70.7 ± 1.3 as, respectively. The errors reported are the SD of the extracted delays over the three measured scans. The measurements were repeated after replacing the amplifier laser crystal and realigning the laser and experiment, and those extracted delays were in agreement with the first set of measurements.

During initial theoretical explorations, up to 11 interacting channels were considered. However, the experimental results are captured accurately with an expansion involving only the X^2^Π*_g_*, a^4^Π*_u_*, A^2^Π*_u_*, and $b^{4}\Sigma_{g}^{-}$ states of $O_{2}^{+}$ . For interpretation of the vibrational-state dependence of the X^2^Π*_g_* state, the four-channel calculations are compared to the uncorrelated, one-channel calculations, capturing just the dynamics of the X^2^Π*_g_* state, to isolate the effects of interchannel coupling.

**Fig. 2.**
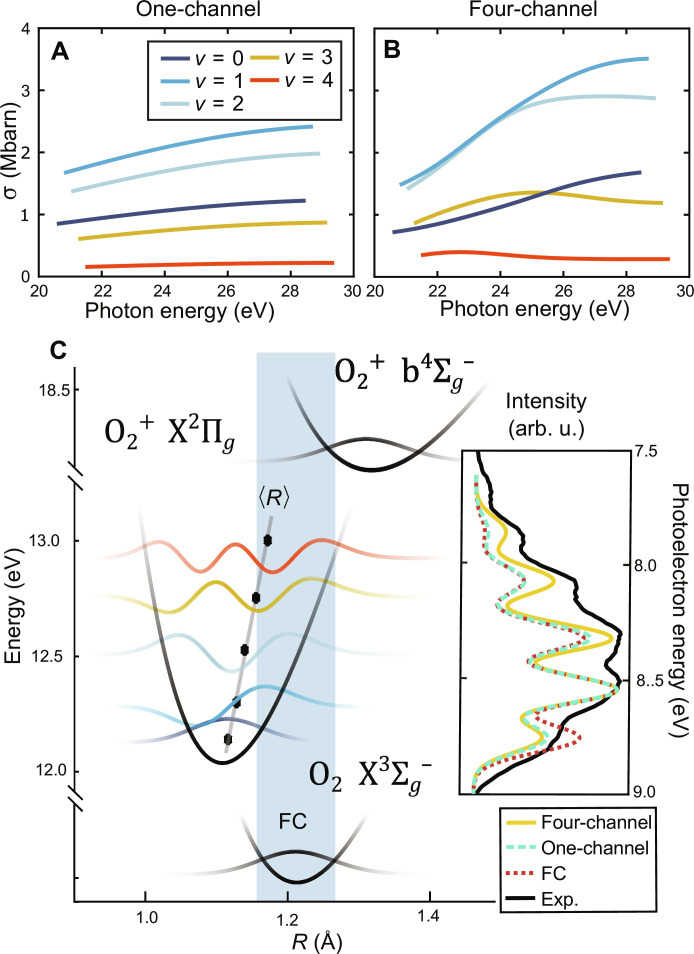
Cross section analysis of the photoionization of O_2_. Vibrational state–resolved photoionization cross sections of the X^2^Π*_g_* state obtained from one-channel (**A**) and four-channel (**B**) calculations. (**C**) The potential energy curves of the relevant states, the different vibrational wavefunctions, their 〈*R*〉, and the Franck-Condon (FC) region (blue highlight). The inset shows a comparison of the X^2^Π*_g_* photoelectron spectrum calculated using the one-channel, four-channel, and FC approximations and compares them to the experimental spectrum. To improve clarity, a slightly higher resolution was chosen in the calculation as compared to the experiment. arb. u, arbitrary units.

Figure 2 (A and B) shows the cross sections and ionization delays for the different vibrational states of the ^2^Π*_g_* state from the one-channel and four-channel calculations. The cross sections in the one-channel calculations (Fig. 2A) closely resemble the simple Franck-Condon (FC) factors that are expected from the adiabatic ionization process, depicted in Fig. 2C. The four-channel calculations predict that, at 21.7 eV (H7), the *v* = 1 and *v* = 2 cross sections are roughly equivalent and that *v* = 3 should be greater than *v* = 0. The inset of Fig. 2C compares the FC, one-channel, four-channel, and experimental photoelectron spectra at 21.7 eV. Over this small energy range, spanning less than 1 eV, it is safe to presume that the instrument response function does not vary considerably, such that the different vibrational-state intensities can be meaningfully compared. The intensities through the vibrational progression are not well captured by the FC factors. They are, however, predicted quite accurately by the four-channel calculations. This indicates a breakdown of the Born-Oppenheimer approximation in this energy region.

**Fig. 3.**
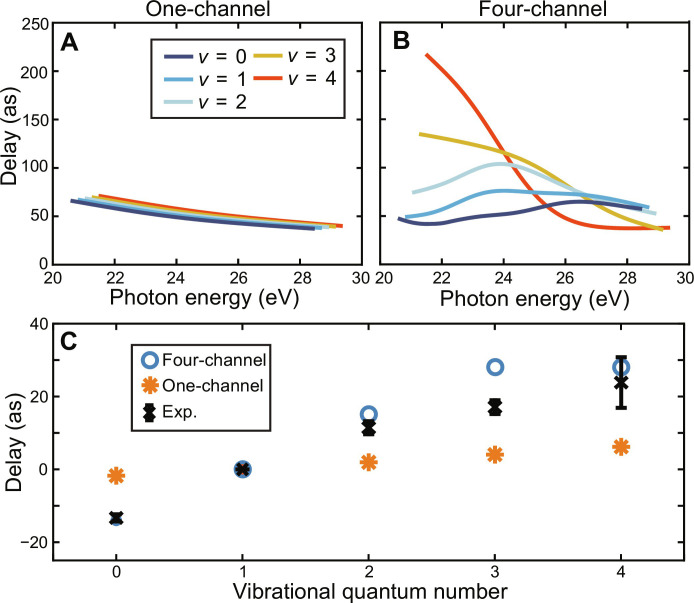
Ionization delay analysis of the photoionization of O_2_. (**A**) One-channel and (**B**) four-channel delay calculations for the different vibrational states of the X^2^Π*_g_* state. (**C**) Comparison of the finite-difference delays derived from the calculations in (A) and (B) to the experimental delays.

Figure 3 shows our delay calculations. The one-channel ionization delay calculations (Fig. 3A) vary minimally with the vibrational quantum number, remaining within about 10 as of one another. In addition, the one-channel delays show virtually no energy dependence besides the general monotonic delay that one expects with increasing kinetic energy. This is in stark contrast to the four-channel calculation (Fig. 3B), where the relative delays of the vibrational states vary by over 200 as and where the state with the largest delay changes multiple times over the calculated energy ranges.

The comparison of the experimental delays to the calculated values is shown in Fig. 3C. As a result of the 6.2-eV separation between harmonics, which is twice as large as in traditional ionization delay measurements, the continuous derivative of the phase of the matrix elements with respect to energy, $\frac{\partial \eta }{\partial E}$ , which gives the delays shown in Fig. 3 (A and B), is not expected to accurately describe the measurements. Instead, the phase difference between harmonics divided by the energy separation between the harmonics, $\frac{\Delta \eta }{\Delta E}$ or finite difference (FD) delay, is the correct metric for comparing the experiment and theory. The one-channel FD delays (orange) displayed in Fig. 3C show almost no variation over the different vibrational states. The four-channel FD delays (blue) vary by ~40 as over the different states, in excellent agreement with the experimental results (black) for all states except for *v* = 3.

## DISCUSSION

**Fig. 4.**
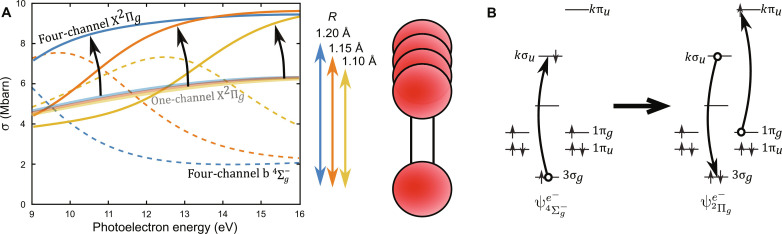
Interpretation of the vibrational state dependence of ionization delays in O_2_. (**A**) The bond-length dependence of the cross section calculations. The different colors demarcate different bond lengths. The one-channel X^2^Π*_g_* cross sections are the solid, semitransparent lines. The four-channel X^2^Π*_g_* cross sections are the solid, nontransparent lines. The dashed, nontransparent lines are for the $b^{4}\Sigma_{g}^{-}$ state, whereby the one-channel and four-channel results are very similar, such that only the four-channel calculations are shown. (**B**) The continuum interchannel coupling, which imprints the $b^{4}\Sigma_{g}^{-}$ shape resonance onto the X^2^Π*_g_* channel.

The origin of the state-dependent delays can be interpreted intuitively by comparing cross section calculations where the O_2_ bond length is varied. For the $b^{4}\Sigma_{g}^{-}$ cross sections, the one- and four-channel calculations show nearly identical results. For clarity, only the four-channel cross section is shown as dashed lines in Fig. 4A. The main feature is a known shape resonance (*5*, *25*), whose central energy shifts with the bond length on average by $-60\;\text{eV}\;{Å}^{-1}$ . This will produce a bond-length–dependent delay in the $b^{4}\Sigma_{g}^{-}$ state.

The one-channel X^2^Π*_g_* cross sections exhibit minimal variation with the bond length, as indicated in the semitransparent lines of Fig. 4A. This confirms that the X^2^Π*_g_* state does not have a shape resonance or shape resonance behavior when considered independently. With the inclusion of the channel coupling, the four-channel X^2^Π*_g_* cross section calculations, shown by the solid lines, exhibit a marked bond-length sensitivity. This distinction is emphasized by the black arrows. This acquired variation with internuclear separation arises from the $b^{4}\Sigma_{g}^{-}$ shape resonance imprinting its shape resonance behavior to the X^2^Π*_g_* through electron correlation. The changes in the cross sections with bond length are accompanied by changes in ionization delay with bond length. To unify this bond-length dependence of delay with the observed vibrational state dependence in the X^2^Π*_g_*, one must consider that each of the different vibrational states has a different 〈*R*〉. Thus, each X^2^Π*_g_* vibrational state experiences a different imprinted shape resonance from the $b^{4}\Sigma_{g}^{-}$ , resulting in each X^2^Π*_g_* vibrational state exhibiting a different ionization delay.

This work introduces high-resolution attosecond interferometry based on a visible driving and dressing wavelength (400 nm). The increased spectral resolution and decreased congestion offered by this approach are exploited to reveal the signature of electron correlations in molecular photoionization delays of O_2_. Specifically, we show that the variation of ∼40 as of the photoionization delays of the X^2^Π*_g_* state across its five lowest vibrational levels is caused by interchannel coupling with the $b^{4}\Sigma_{g}^{-}$ channel, which features a shape resonance that strongly depends on the internuclear separation. This pronounced sensitivity of the energetic position of the shape resonance in the $b^{4}\Sigma_{g}^{-}$ channel on the bond length translates to the large variation of the photoionization delays of the X^2^Π*_g_* state with the vibrational quantum number *v*. More generally, this work showcases the benefits of using visible wavelengths as both driving and dressing wavelength in attosecond interfeometry, which will be particularly beneficial for the study of complex systems, such as clusters (*26*), liquids (*27*), and solvated systems.

## MATERIALS AND METHODS

### Experimental setup

A 1-kHz, Femtopower V Pro CEP amplifier produces the light for these experiments. The integrated acousto-optic programmable dispersive filter shapes a 45-nm (full width at half maximum), 800-nm central-wavelength spectrum at the output of the amplifier. A polarizer and half-wave plate attenuator adjust the total beam power to ~1.2 W measured after the transmissive grating compressor. The 35-fs light passes through a 0.5-mm beta-barium-borate crystal to generate 0.3 mJ of spectrally narrow 55-fs light, centered at 400 nm. A 0.50-m focal length lens focuses the 400-nm light into a differentially pumped, 4-mm diameter gas cell filled with ∼30 mbar of Ar. The gas pressure, target position, and 800-nm compression are adjusted to maximize the flux in harmonics seven (21.7 eV) and nine (27.9 eV). The 400-nm light and the generated harmonics copropogate through an adjustable iris that tunes the dressing field strength to maximize the sideband amplitude with minimal tunneling ionization. In our experience, tunneling ionization saturates the count rate and blurs the spectral resolution before any 4ω oscillations are observed. The sideband amplitude is also rather small compared to the main peaks. This is expected on the basis of the scaling laws for the dressing efficiency being proportional to λ^2^ (*28*). Together, the absence of 4ω oscillations and low sideband amplitudes ensures that the measurements are taken with the dressing intensity in the perturbative regime.

The copropogating 400-nm light and XUV traverse a series of transmissive optics to filter them spectrally while maintaining passive temporal stability. A 2-μm cellulose pellicle was etched using acetone to create a 2.5- to 3.0-mm-wide aperture, which was inserted into the beam. The XUV light passes through the hole in the center, but, elsewhere, the pellicle suppresses any unwanted XUV from the dressing field region. This prevents a static sideband amplitude in the measurements that arises from XUV reflecting off the outer mirror with the dressing field. The XUV light that passes through the center is filtered with a 3.0-mm-diameter, 200-nm-thick, aluminum filter (Lebow). This suppresses the 400-nm light from the center of the beam, which can otherwise produce a 1ω component in the interferogram. This 3-mm-wide filter can be replaced with a 10-mm-wide filter that suppresses all of the dressing light to record an XUV-only spectrum. After spectral filtering, the dual-mirror assembly, which has a delay stability below 10 as (*29*), uses mirrors with optimized reflectivity between 20 and 30 eV to focus the light into the interaction region. Here, the XUV and dressing light ionize the sample and the dressed photoelectrons pass through a 500-μm skimmer before entering a 1.5-m magnetic-bottle time-of-flight spectrometer.

### Theoretical and computational process

To explain the measurements, we compare them to theoretical calculations of the time delays that used Born-Oppenheimer photoionization vibronic wave functions $\Psi_{\alpha ,v_{\alpha },{\vec{k}}_{\alpha }}^{\left(-\right)}$ of the form (*30*)

$$\Psi_{\alpha ,v_{\alpha },k_{\alpha }}^{\left(-\right)}\left(\vec{r},R\right)=\sum_{l_{\alpha },m_{\alpha }}\psi_{\alpha ,l_{\alpha },m_{\alpha },\in_{\alpha }}^{\left(-\right)}\left(\vec{r};R\right)X_{v\alpha }\left(R\right)Y_{l_{\alpha },m_{\alpha }}^{*}\left({\hat{k}}_{\alpha }\right)$$ (1)

where α indicates the final ion state, χ_*v*α_(*R*) is the final vibrational state of the ion and ${\vec{k}}_{\alpha }$ is an asymptotic momentum of the photoelectron with energy $\epsilon_{\alpha }=\frac{1}{2}k_{\alpha }^{2}$ . Note that $\psi_{\alpha ,l_{\alpha },m_{\alpha },\epsilon_{\alpha }}^{\left(-\right)}\left(\vec{r};R\right)$ is the fixed-nuclei photoionization electronic wave function computed at *R*. With final state $\Psi_{\alpha ,v_{\alpha },{\vec{k}}_{\alpha }}^{\left(-\right)}$ , the transition dipole matrix elements are computed using

$$I_{\alpha ,v_{\alpha },\vec{\mu }}\left({\vec{k}}_{\alpha }\right)=\langle \Psi_{\alpha ,v_{\alpha },{\vec{k}}_{\alpha }}^{\left(-\right)}|\vec{\mu }\cdot \vec{r}{|\Psi_{0}\rangle }_{\;\vec{r},R}$$ (2)

The time delay for fixed orientation is obtained as the derivative of the argument of this matrix element. To compare with the experiment, the time delay is averaged over all emission directions $\hat{k}$ and polarization directions $\hat{\mu }$ weighted by the differential photoionization cross sections (*31*).

The dipole matrix elements in Eq. 2 were computed using the multichannel Schwinger configuration interaction method (*32*, *33*). The full *N*-electron–ionized state was represented by a close-coupling expansion containing a sum of the products of (*N − 1*)–electron ion state wave functions times one-electron photoelectron wave functions. At each internuclear distance *R*, a set of orbitals was computed with an aug-cc-pVTZ basis (*34*, *35*) using MOLPRO (*36*, *37*) with a valence complete active space self-consistent field (CASSCF) description of the ground state keeping the 1σ*_g_* and 1σ*_u_* core orbitals doubly occupied. The *N −* 1 electron ion states were then obtained from a complete active space configuration interaction calculation using the orbitals from the ground-state valence CASSCF calculation.

To compute the integral over *R*, indicated in Eq. 2, the photoionization calculations were performed on a grid of points from *R* = 0.80 Å to *R* = 1.50 Å with steps of 0.05 Å. The values of the matrix elements were interpolated using a cubic spline onto a finer radial grid for accurate integrals over *R*. The potential energy curves for the ground state and four ion states were Morse potentials constructed from experimental spectroscopic ω*_e_*, ω*_e_x_e_*, and *r_e_* values (*38*). In the close-coupling photoionization calculations, the ionization potentials of the four ion states were adjusted to agree with the Morse potential energy curves, which were shifted in energy to agree with the experimental vertical binding energies (*39*).
